# A conservation checklist of the amphibians and reptiles of Mexico City, with comparisons with adjoining states

**DOI:** 10.3897/zookeys.951.52578

**Published:** 2020-07-22

**Authors:** Julio A. Lemos-Espinal, Geoffrey R. Smith

**Affiliations:** 1 Laboratorio de Ecología-UBIPRO, FES Iztacala UNAM, Avenida los Barrios 1, Los Reyes Iztacala, Tlalnepantla, edo. de México, 54090, México FES Iztacala UNAM Tlalnepantla Mexico; 2 Department of Biology, Denison University, Granville, Ohio 43023, USA Denison University Granville United States of America

**Keywords:** amphibians, frogs, herpetofauna, lizards, reptiles, salamanders, snakes, turtles

## Abstract

Mexico City houses one of the most populous urban areas of the world, and the modification of its natural habitat likely influences the biological diversity found there. In particular, amphibians and reptiles are likely affected by these modifications. Herein, we present an updated list of the species of amphibians and reptiles that inhabit Mexico City. Mexico City harbors 65 species of amphibians and reptiles, which represent 21 families and 33 genera. These include 18 species of amphibians (nine anurans and nine salamanders) and 47 species of reptiles (14 lizards, 30 snakes [one introduced], and three turtles [one introduced]). Forty-eight of the amphibian and reptile species in Mexico City are endemic to Mexico, with two endemic to Mexico City. The most diverse region of Mexico City is the Forests and Ravines region, which is home to 43 species. Eleven species of amphibians and reptiles in Mexico City are IUCN listed, 16 are placed in a protected category by SEMARNAT (Secretaria del Medio Ambiente y Recursos Naturales), and 27 species are categorized as high risk by the EVS (Environmental Viability Score). Mexico City shares almost 94% of its species with the State of Mexico.

## Introduction

**Figure 1.**
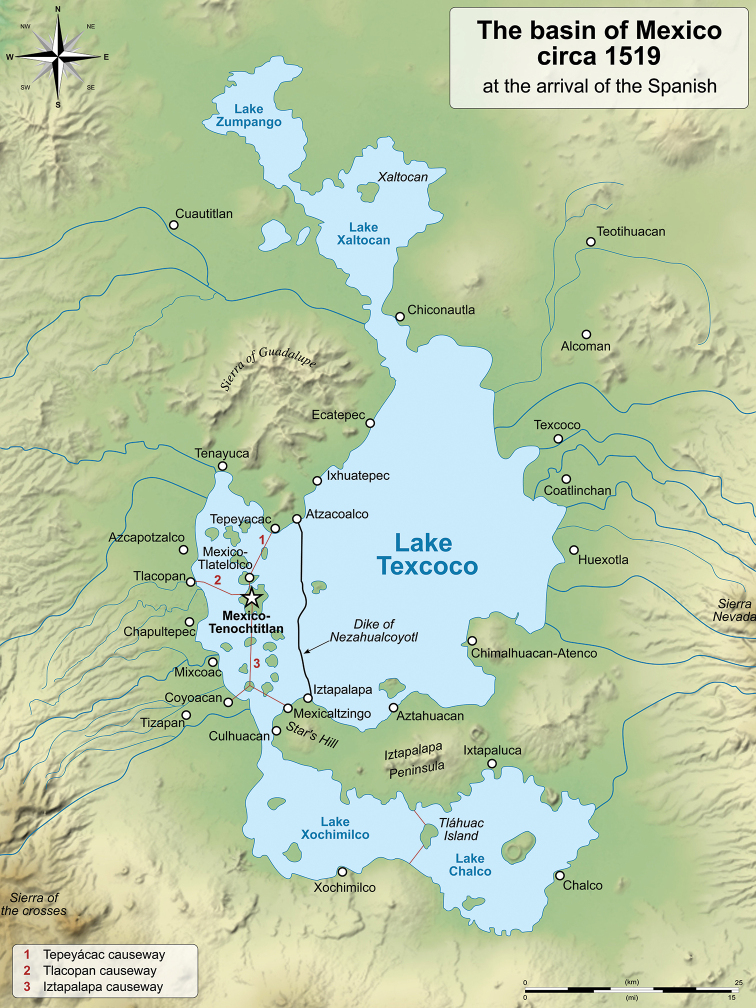
Basin of Mexico, circa 1519. Source: https://upload.wikimedia.org/wikipedia/commons/9/99/Basin_of_Mexico_1519_map-en.svg

Since pre-Hispanic times the Basin of Mexico, upon which Mexico City (formerly Mexico, Distrito Federal) sits, caught the attention of the inhabitants of central Mexico. This large lake surrounded by fertile land was the location of important human settlements that, at the arrival of the Spaniards, were represented mainly by Tenochtitlan, which along with a large number of villages located around the basin reached over a million inhabitants (Wikipedia: https://es.wikipedia.org/wiki/Ciudad_de_M%C3%A9xico – accessed 27 December 2019). At the arrival of the Spaniards, in 1519, the basin was occupied by a well-developed civilization whose economy revolved around the Chinampas that surrounded the lake (Fig. 1). With the Spanish conquest of Mexico, cattle were introduced to the basin and a radical transformation began, including the drying of the Basin and the felling of the forests that surrounded it. The population around the Basin began to decline to such extent that at the end of the 18^th^ century the number of inhabitants in Mexico City was only 120,000. It was not until the middle of the 20^th^ century that the population explosion began creating diverse and complex problems that today overwhelm Mexico City (Imaz 1989). Currently the population of Mexico City is approximately 8.9 million inhabitants; however, including the metropolitan area of the Valley of Mexico, which extends over the Basin of Mexico, the population totals 22 million inhabitants, the ninth most populated urban area in the world, and the largest in the Americas (Wikipedia: https://es.wikipedia.org/wiki/Ciudad_de_M%C3%A9xico – accessed 27 December 2019).

The urban area of Mexico City covers almost the whole northern half of Mexico City *sensu lato* (previously Distrito Federal) and is rapidly expanding (e.g., Hernández-Flores et al. 2017), including through illegal development (Rodríguez López et al. 2017). The southern half of Mexico City *sensu lato* is occupied by mountains and ravines covered by extensive forests and grasslands, some of them highly fragmented by housing developments and cultivated fields. The air pollution is such that the governments of Mexico City and the State of Mexico created a program at the end of 1989 that limits motor vehicle use in the city. However, air pollution continued to increase despite the initial restrictions, and currently there are increased limitations on vehicle use. Spaces for new residential development within the city are fewer and the population continues to grow and demand more resources, increasing the production of waste, the emission of greenhouse gases, the number of fires, the illegal occupation of protected areas, and the depletion of available natural resources (Santibáñez-Andrade et al. 2015; SEMARNAT 2016; Heider et al. 2018). Increased urbanization around Mexico City proper has also affected rivers and streams (Caro-Borrero et al. 2016). This considerable modification of the natural habitat exerts a constant pressure on the biological diversity of Mexico City. Amphibians and reptiles are strongly affected by these changes, and there are species whose presence in Mexico City is known only from their original records (e.g., *Geophis bicolor* and *G. petersi*), or whose conservation status is quite tenuous (e.g., *Eleutherodactylus grandis*, *Rana tlaloci*, *Ambystoma mexicanum*, and *Crotalus transversus*).

Herein, we present an updated list of amphibians and reptiles that inhabit Mexico City in an effort to disseminate important information about its herpetofauna to help in their conservation. Previous recent efforts to catalog the herpetofauna of Mexico City reported a list of 18 species of amphibians and 39 species (one of them introduced) of reptiles from Mexico City (García-Vázquez and Méndez-de la Cruz 2016; García-Vázquez et al. 2016).

### Physiographic characteristics

**Figure 2.**
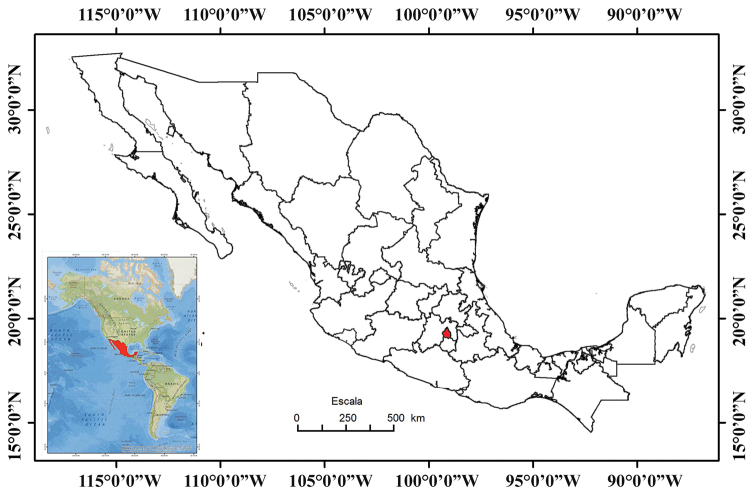
Map of Mexico with Mexico City shown in red (modified from INEGI 2018).

**Figure 3.**
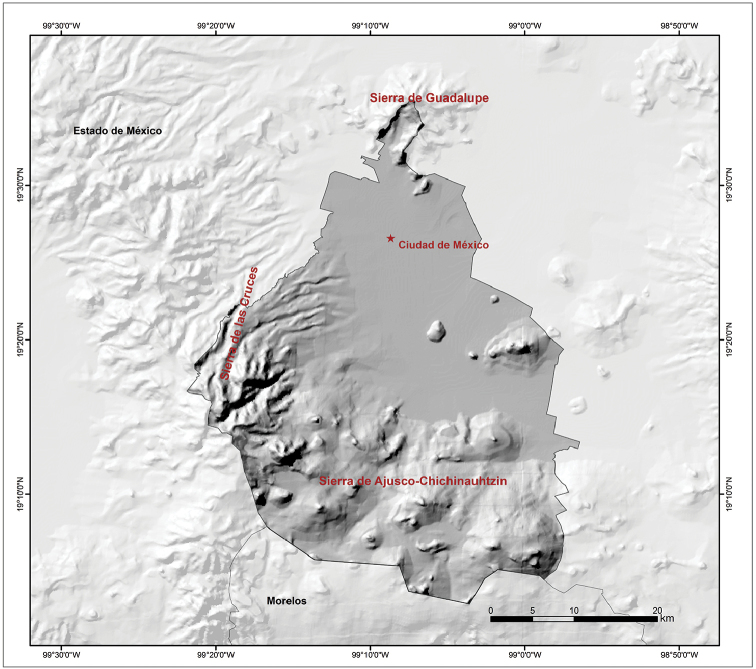
Topographical map of Mexico City, Mexico (Source: CONABIO 1997).

Mexico City is one of 32 federal entities in Mexico. It is the capital of the country. It is located between 19°35'34"N and 19°2'54"N, and 98°56'25"W and 99°2154'W. It is bordered by the State of Mexico to the north, east, and west, and by Morelos to the south (Fig. 2). It covers 1,485 km^2^, which represents 0.1% of the total area of Mexico. The urban area of Mexico City is in the Valley of Mexico, a large valley in the high plateaus in the center of Mexico, at an altitude of 2,240 m (Figs 2, 3; INEGI 2017; https://en.wikipedia.org/wiki/Mexico_City – accessed 17 December 2019).

**Figure 4.**
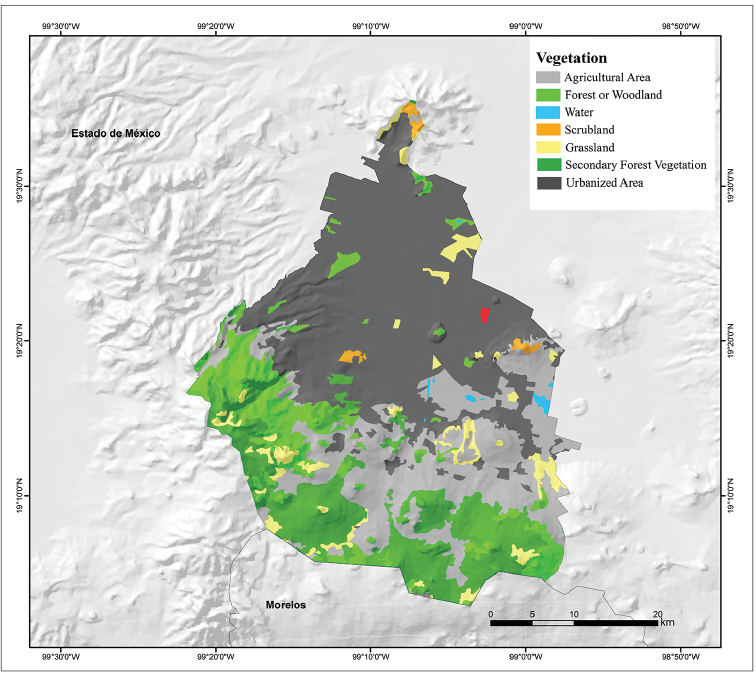
Vegetation map of Mexico City, Mexico (modified from Dirección General de Geografía; INEGI 2016).

**Figure 5.**
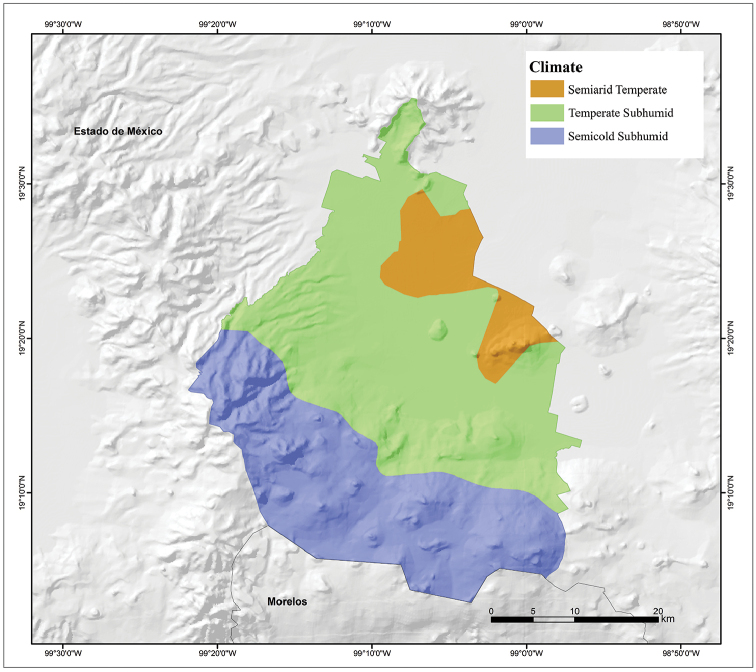
Climate map of Mexico City, Mexico (modified from García and Comisión Nacional para el Conocimiento y Uso de la Biodiversidad 1998).

The topography of Mexico City is highly variable, including an extensive high plateau in the northern half of the city, where the urban area sits, and mountains and volcanoes that reach up to 3,930 m of altitude (Volcán Ajusco) that surround the urban area mainly on its southern and western sides (Fig. 3). Mexico City is located in the physiographic province of the Neovolcanic Axis, sub-province of Lagos and Volcanes of Anahúac. Mexico City is divided into an urban development area, usually referred to as urban land (41% of the territory, mostly on the northern half of the city), and an ecological conservation area, referred to as conservation land (59% of the territory, mostly on the southern half of the city). Urban land primarily consists of the urbanized plain area of the city, whereas conservation land includes areas with natural ecosystems (Reygadas-Prado 2016). Natural vegetation is primarily distributed in the conservation land, and in general terms it is represented by forests (55.6%), agricultural areas (35.8%), grasslands (7.17%), and scrublands (1.43%) (Fig. 4; INEGI 2017). According to the Köppen climate classification modified by García (1998), the climate of Mexico City is broadly divided into Subhumid Temperate with summer rains, covering much of the city (Fig. 5). A Semicold Subhumid climate with summer rains is present in the highest parts of the mountains that surround the city, from the west-central part running diagonally to the southeastern end on the border with Morelos; and Semiarid Temperate with summer rains present in the east-northeast end of the city, including the Sierra de Santa Catarina in the eastern part of the city and a considerable portion of the northeastern end of the city in the boroughs of Gustavo A. Madero, Venustiano Carranza, and Iztacalco (Fig. 5).

**Figure 6.**
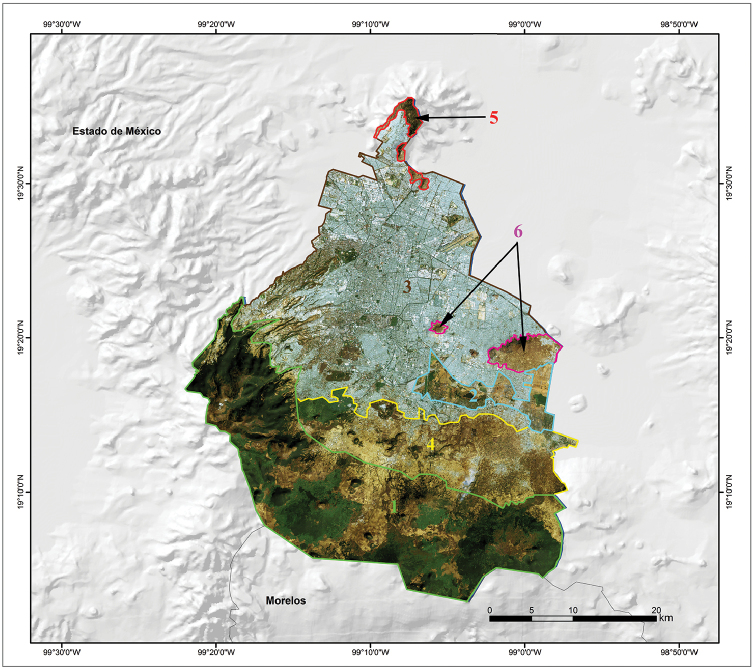
Satellite map showing the topographic features of Mexico City (source: Comisión Nacional para el Conocimiento y Uso de la Biodiversidad 2003) and Regions of Mexico City according to Reygadas-Prado (2016): 1) Forests and Ravines; 2) Wetlands of Xochimilco and Tláhuac; 3) Urban Parks and Gardens; 4) Mountains of Xochimilco and Milpa Alta; 5) Sierra de Guadalupe; 6) Sierra de Santa Catarina.

Reygadas-Prado (2016) provided a regionalization of Mexico City based on biophysical characteristics that consists of six regions. The Forests and Ravines region is made up of the largest and best-preserved forest massifs and ravines found in the south and southwest of the city, occupying an area of 532.4 km^2^. The principal forests in this region are oak, pine-oak, and pine forests, with some cloud forest relics in the vicinity of the Dínamos de Contreras. The Wetlands of Xochimilco and Tláhuac region is found in the Chinampas area of Xochimilco, San Gregorio, San Luis Tlaxialtemanco, Tláhuac and the lowlands of Tláhuac, occupying an area of 60.3 km^2^. This region is located in the east-central parts of the city. The Urban Parks and Gardens region includes parks, gardens, forests, protected natural areas, and areas of environmental value located on urban land, and occupies an area of 607.3 km^2^. It is found in the northern half of the city, except for the Sierra de Guadalupe, and two areas in the central and eastern parts of the city that represent the Sierra de Santa Catarina region. This region includes the urbanized area of Mexico City. The Mountains of Xochimilco and Milpa Alta region is found between the Urban Parks and Gardens and the Forests and Ravines regions, and it occupies an area of 237.5 km^2^. It runs from west-central to eastern Mexico City. It consists of an area of forests fragmented by housing developments; however, important areas of oak, pine-oak, and pine forests are located in this region. The Sierra de Guadalupe region is located in northern Mexico City and includes the protected natural areas of Sierra de Guadalupe, La Armella, and Tepeyac National Park and occupies an area of 12.9 km^2^. Its characteristic vegetation is xerophilous scrub. The Sierra de Santa Catarina region, located in eastern and central Mexico City, occupies an area of 31.4 km^2^. The vegetation here is similar to the Sierra de Guadalupe, with an important area of grassland and a greater number of tree species for reforestation (Fig. 6; Reygadas-Prado 2016).

## Materials and methods

We compiled our list of amphibians and reptiles for Mexico City using our fieldwork, a thorough examination of the literature, records from VertNet.org and Servicio de Descarga de Ejemplares del Sistema Nacional de Información sobre Biodiversidad (SNIB-CONABIO): Amphibians Ciudad de México and Reptiles Ciudad de México data bases. We included species in the list if they had confirmed records, either by direct observation, literature report, or through documented museum records or vouchers. We follow Frost (2020) and AmphibiaWeb (2020) for amphibian names and Uetz and Hošek (2019) for reptile names.

We did not include some of the species in the Rüthling collection (AMNH) for Mexico City, as that collection includes specimens from a German School Collection, from Mexico City. These were donated to Rüthling, namely *Sonora michoacanensis mutabilis* Stickel, 1943 (paratypes: AMNH R-19714-6), *Trimorphodon tau tau* (Cope, 1870) (AMNH R-19718), *Micrurus elegans elegans* (Jan, 1858) (AMNH R-19720), and *Thamnophis proximus* (Say, 1823) (AMNH R-19809-10), but do not occur in Mexico City. In addition, some of the specimens in the German School Collection obtained by Rüthling were most likely not collected in Mexico City (Zweifel 1959). However, some of the specimens in the German School Collection donated to Rüthling could have been collected in Mexico City (e.g., *Senticolis triaspis* (Cope, 1866) [AMNH R-19838]), as there are two more records of this species in other collections: BMNH 1868.4.7.16 collected by Doorman and reported by Günther (1894: 115) and ENCB-IPN 7398 from San Gregorio, Xochimilco). Other specimens from the Rüthling collection donated by the German School are likely to occur in Mexico City (e.g., *Tantilla bocourti* (Günther, 1895) [AMNH R-19735]), but as no other records of the species are available, we decided not to include this species in our list.

We created species accumulation curves for the total herpetofauna, as well as amphibians and reptiles separately, using the year of the first recorded observation for each species. Such species accumulation curves likely provide a good estimate of potential herpetofaunal richness (see Raxworthy et al. 2012). In addition, we recorded the conservation status of each species based on the IUCN Red List 2019-2 (IUCN 2019), SEMARNAT (2019), and Environmental Vulnerability Scores from Wilson et al. (2013a, b) and Johnson et al. (2015). We determined the number of overlapping species with the two states that neighbor Mexico City using recent state lists (State of Mexico, Lemos-Espinal and Smith in press; Morelos, Lemos-Espinal and Smith in 2020).

## Results and discussion

**Figure 7.**
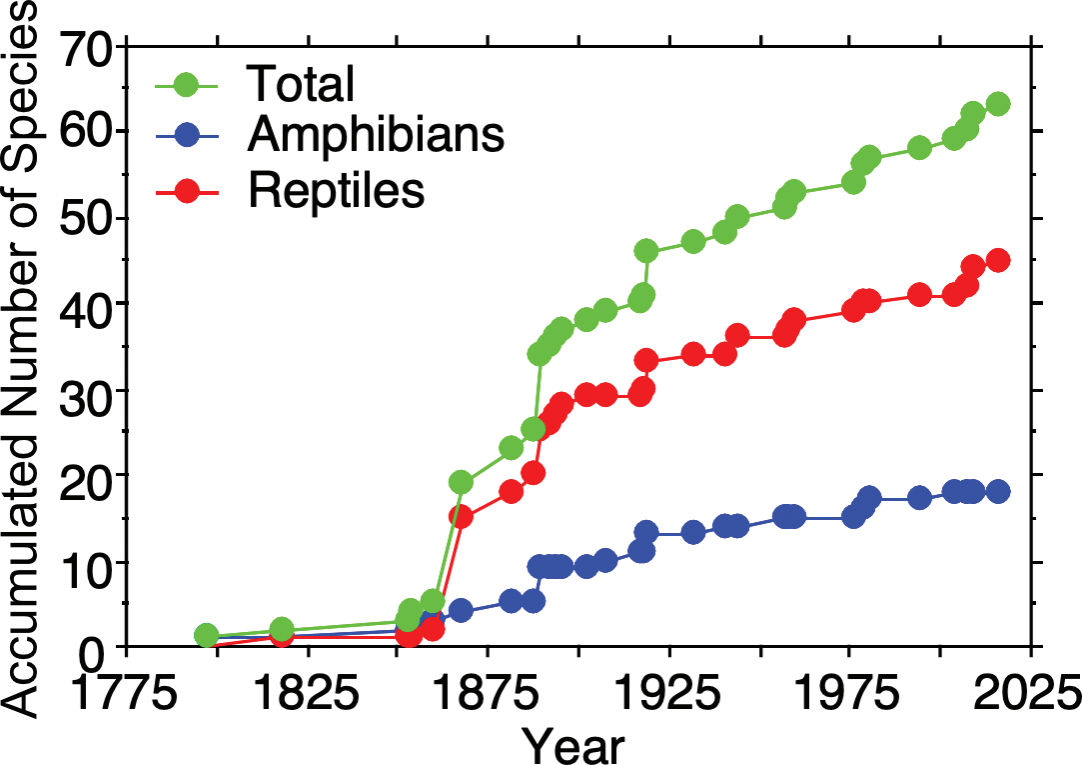
Species accumulation curves for total herpetofauna of Mexico City, Mexico, as well as separately for amphibians and reptiles.

Mexico City harbors 65 species of amphibians and reptiles, which represent 21 families (two introduced: Typhlopidae Merrem, 1820 and Emydidae [Rafinesque, 1815]) and 33 genera (two introduced: *Indotyphlops* Hedges, Marion, Lipp, Marin & Vidal, 2014 and *Trachemys* Agassiz, 1857) (Tables 1, 2). These include 18 species of amphibians (nine anurans and nine salamanders) and 47 reptiles (14 lizards, 30 snakes [one introduced], and three turtles [one introduced]). It appears that this list of amphibian and reptile species is likely fairly complete given the shape of the species accumulation curves (Fig. 7). The species accumulation curves indicate a rapid accumulation of species in the late 1800s, followed by a slow gradual increase in species throughout the 20^th^ and early 21^st^ centuries, with a leveling off in recent years.

**Table 1.** Amphibians and reptiles of Mexico City with distributional and conservation status. Region: (1 = Forests and Ravines; 2 = Wetlands of Xochimilco and Tláhuac; 3 = Urban Parks and Gardens; 4 = Mountains of Xochimilco and Milpa Alta; 5 = Sierra de Guadalupe; 6 = Sierra de Santa Catarina) according to Reygadas-Prado (2016). Species with asterisk (*Geophis bicolor* and *G. petersii*), are records without a specific locality, assigned to a region that we consider the best fit, and species with a question mark (*Lampropeltis polyzona* and *Micrurus tener*) are records without a specific locality representing species with a wide range of habitat use, such that is not possible to assign them to a specific region. IUCN Status: (DD = Data Deficient; LC = Least Concern, VU = Vulnerable, NT = Near Threatened; EN = Endangered; CR = Critically Endangered; NE = not Evaluated) according to the IUCN Red List (The IUCN Red List of Threatened Species, Version 2019-2 (www.iucnredlist.org; accessed 29 November 2019); conservation status in Mexico according to SEMARNAT (2019): (P = in danger of extinction, A = threatened, Pr = subject to special protection, NL = not listed); Environmental Vulnerability Score: (EVS – the higher the score the greater the vulnerability: low (L) vulnerability species (EVS of 3–9); medium (M) vulnerability species (EVS of 10–13); and high (H) vulnerability species (EVS of 14–20) from Wilson et al. (2013a,b) and Johnson et al. (2015); Global Distribution: 0 = Endemic to Mexico City; 1 = Endemic to Mexico; 2 = Shared between the US and Mexico; 3 = widely distributed from Mexico to Central or South America; 4 = widely distributed from the US to Central or South America; IN = introduced to Mexico City. Date in which the first record appeared; and Source of the first record.

	Region	IUCN	SEMARNAT	EVS	Global	Year	Source
**Class Amphibia**							
**Order Anura**							
**Family Bufonidae Gray, 1825**							
*Anaxyrus compactilis* (Wiegmann, 1833)	1, 2, 3, 4, 5	LC	NL	H (14)	1	1890	Herrera 1890
**Family Craugastoridae Hedges, Duellman, & Heinecker, 2008**							
*Craugastor augusti* (Dugès, 1879)	5	LC	NL	L (8)	2	1981	Méndez de la Cruz et al. 1992
**Family Eleutherodactylidae Lutz, 1954**							
*Eleutherodactylus grandis* (Dixon, 1957)	3	CR	Pr	H (18)	0	1957	Dixon 1957
**Family Hylidae Rafinesque, 1815**							
*Dryophytes arenicolor* (Cope, 1886)	1, 3	LC	NL	L (7)	2	1919	AMNH A-13254
*Dryophytes eximius* (Baird, 1854)	1, 2, 3, 4, 5	LC	NL	M (10)	1	1853	USNM 3248
*Dryophytes plicatus* (Brocchi, 1877)	1, 3	LC	A	M (11)	1	1917	MCZ-A 17702
**Family Ranidae Batsch, 1796**							
*Rana montezumae* Baird, 1854	2, 3, 4, 5	LC	Pr	M (13)	1	1854	Baird 1854
*Rana tlaloci* Hillis & Frost, 1985	1, 2, 3, 5	CR	P	H (15)	1	1919	AMNH A-12214
**Family Scaphiopodidae Cope, 1865**							
*Spea multiplicata* (Cope, 1863)	1, 2, 3, 4	LC	NL	L (3)	2	1890	Herrera 1890
**Order Caudata**							
**Family Ambystomatidae Gray, 1850**							
*Ambystoma altamirani* Dugès, 1895	1, 3	EN	A	M (13)	1	1908	Gadow 1908
*Ambystoma mexicanum* (Shaw & Nodder, 1798)	2	CR	P	H (15)	0	1798	Shaw and Nodder 1798
*Ambystoma velasci* Dugès, 1888	5	LC	Pr	M (10)	1	1882	USNM 12721
**Family Plethodontidae Gray, 1850**							
*Aquiloeurycea cephalica* (Cope, 1865)	1, 3	NT	A	H (14)	1	1941	AMNH A-52026
*Chiropterotriton orculus* (Cope, 1865)	1, 3, 4	VU	NL	H (18)	1	1890	Herrera 1890
*Isthmura belli* (Gray, 1850)	1, 3	VU	A	M (12)	1	1868	BMNH 1868.4.7.37-38
*Pseudoeurycea altamontana* (Taylor, 1939)	1	EN	Pr	H (17)	1	2004	MZFC 23438
*Pseudoeurycea leprosa* (Cope, 1869)	1, 3, 4	LC	A	H (16)	1	1890	Herrera 1890
*Pseudoeurycea tlilicxitl* Lara-Góngora, 2003	1	EN	NL	H (17)	1	1979	CNAR 3682
**Class Reptilia**							
**Suborder Lacertilia**							
**Family Anguidae Gray, 1825**							
*Barisia imbricata* (Wiegmann, 1828)	1, 2, 3, 4, 5, 6	LC	Pr	H (14)	1	1868	BMNH 1868.4.7.45-47
**Family Phrynosomatidae Fitzinger, 1843**							
*Phrynosoma orbiculare* (Linnaeus, 1758)	1, 3, 4, 5, 6	LC	A	M (12)	1	1818	MVZ 43510
*Sceloporus aeneus* Wiegmann, 1828	1	LC	NL	M (13)	1	1918	AMNH R-15492
*Sceloporus anahuacus* Lara-Góngora, 1983	1	LC	NL	H (15)	1	1976	Lara-Góngora 1983
*Sceloporus grammicus* Wiegmann, 1828	1, 2, 3, 4, 5, 6	LC	Pr	L (9)	2	1892	USNM 18994
*Sceloporus mucronatus* Cope, 1885	1	LC	NL	M (13)	1	1944	AMNH R-65701
*Sceloporus palaciosi* Lara-Góngora, 1983	1, 4	LC	NL	H (15)	1	1979	MZFC 790
*Scelopours scalaris* Wiegmann, 1828	2, 3, 5, 6	LC	NL	M (12)	1	1896	USNM 46877
*Sceloporus spinosus* Wiegmann, 1828	5, 6	LC	NL	M (12)	1	1882	USNM 12720
*Sceloporus sugillatus* Smith, 1942	1	LC	NL	H (16)	1	2008	Mendoza-Hernández et al. 2008
*Sceloporus torquatus* Wiegmann, 1828	1, 2, 3, 4, 5, 6	LC	NL	M (11)	1	1882	USNM 12719
**Family Scincidae Gray, 1825**							
*Plestiodon brevirostris* (Günther, 1860)	1	LC	NL	M (11)	1	?	FMNH w/o number
*Plestiodon copei* (Taylor, 1933)	1	LC	Pr	H (14)	1	1958	ENCB 810
**Family Teiidae Gray, 1827**							
*Aspidoscelis gularis* (Baird & Girard, 1852)	4, 5, 6	LC	NL	L (9)	4	1919	AMNH R-14221
**Suborder Serpentes**							
**Family Colubridae Oppel, 1811**							
*Conopsis biserialis* (Taylor & Smith, 1942)	3	LC	A	M (13)	1	1960	ENCB 124
*Conopsis lineata* (Kennicott, 1859)	1, 3, 4	LC	NL	M (13)	1	1890	Herrera 1890
*Conopsis nasus* (Günther, 1858)	3, 5	LC	NL	M (11)	1	1903	BMNH 1903.9.30.200
*Lampropeltis polyzona* Cope, 1860	?	LC	NL	L (7)	1	1868	BMNH 1868.4.7.4
*Pituophis deppei* (Dumeril, 1853)	1, 2, 3, 4, 5, 6	LC	A	H (14)	1	1868	BMNH 1868.4.7.38
*Pituophis lineaticollis* (Cope, 1861)	1	LC	NL	L (8)	3	1932	FMNH 106582
*Salvadora bairdi* Jan & Sordelli, 1860	1, 3, 5, 6	LC	Pr	H (15)	1	1919	AMNH R-19528
*Senticolis triaspis* (Cope, 1866)	2	LC	NL	L (6)	4	1868	BMNH 1868.4.7.16
*Tantilla calamarina* Cope, 1866	1	LC	Pr	M (12)	1	1919	AMNH R-19750
**Family Dipsadidae Bonaparte, 1838**							
*Diadophis punctatus* (Linnaeus, 1766)	1, 2, 3, 5	LC	NL	L (4)	2	1868	Günther 1868
*Geophis bicolor* Günther, 1868	1*	DD	Pr	H (15)	1	1868	Günther 1868
*Geophis petersii* Boulenger, 1894	1*	DD	Pr	H (15)	1	1894	Boulenger 1894
*Rhadinaea laureata* (Günther, 1868)	1,3,6	LC	NL	M (12)	1	1868	Günther 1868
*Rhadinaea taeniata* (Peters, 1863)	1	LC	NL	M (13)	1	1868	BMNH 1868.4.7.13-14
**Family Elapidae Boie, 1827**							
*Micrurus tener* Baird & Girard, 1853	?	LC	NL	M (11)	2	1868	BMNH 1868.4.7.5
**Family Leptotyphlopidae Stejneger, 1892**							
*Rena dulcis* Baird & Girard, 1853	3	LC	NL	M (13)	2	2009	Méndez de la Cruz et al. 2009
**Family Natricidae Bonaparte, 1838**							
*Storeria storerioides* (Cope, 1866)	1, 3, 4	LC	NL	M (11)	1	1868	BMNH 1868.4.7.15
*Thamnophis cyrtopsis* (Kennicott, 1860)	1	LC	A	L (7)	4	1890	Herrera 1890
*Thamnophis eques* (Reuss, 1834)	1, 3, 5, 6	LC	A	L (8)	2	1860	Kennicott 1860
*Thamnophis melanogaster* (Wiegmann, 1830)	2, 3	EN	A	H (15)	1	1882	USNM 12726
*Thamnophis pulchrilatus* (Cope, 1885)	1	LC	NL	H (15)	1	1890	Herrera 1890
*Thamnophis scalaris* Cope, 1861	1, 3, 5	LC	A	H (14)	1	1890	Herrera 1890
*Thamnohis scaliger* (Jan, 1863)	1, 2, 3, 4	VU	A	H (15)	1	1868	BMNH 1868.4.7.10
**Family Typhlopidae Merrem, 1820**							
*Indotyphlops braminus* (Daudin, 1803)	3	IN	NA	NA	NA	1995	CNAR 11281
**Family Viperidae Oppel, 1811**							
*Crotalus aquilus* Klauber, 1952	5	LC	Pr	H (16)	1	2016	García-Vázquez and Méndez de la Cruz 2016
*Crotalus molossus* Baird & Girard, 1853	3, 4, 5, 6	LC	Pr	L (8)	2	1868	BMNH 1868.4.7.2
*Crotalus polystictus* (Cope, 1865)	2	LC	Pr	H (16)	1	1890	Herrera 1890
*Crotalus ravus* Cope, 1865	1, 2, 3, 4, 5, 6	LC	A	H (14)	1	1944	UMMZ 99847
*Crotalus transversus* Taylor, 1944	1	LC	P	H (17)	1	?	ROM 47094
*Crotalus triseriatus* (Wagler, 1830)	1, 2, 3, 4	LC	NL	H (16)	1	1868	BMNH 1946.1.17.70
**Order Testudines**							
**Family Emydidae (Rafinesque, 1815)**							
*Trachemys venusta* (Gray, 1855)	3	IN	NA	NA	NA	2009	Méndez de la Cruz et al. 2009
**Family Kinosternidae Agassiz, 1857**							
*Kinosternon hirtipes* (Wagler, 1830)	2,5	LC	Pr	M (10)	2	1888	Dugès 1888
*Kinosternon integrum* LeConte, 1854	2	LC	Pr	M (11)	1	1888	Dugès 1888

The two introduced species are Brahminy Blindsnake, *Indotyphlops braminus* (Daudin, 1803), and Huastecan Slider, *Trachemys venusta* (Gray, 1855). Two of the 63 native species are endemic to Mexico City: Great Piping Frog, *Eleutherodactylus grandis* (Dixon, 1957), and Axolotl, *Ambystoma mexicanum* (Shaw & Nodder, 1798). The latter species originally inhabited lakes in the State of Mexico and Mexico City but is currently only known from the remnants of Lake Xochimichilco (Frost 2020). The most species-rich amphibian family is Plethodontidae Gray, 1850, and the most species-rich reptile family is Phrynosomatidae Fitzinger, 1843 (Table 1).

We compiled a list of eight species (six amphibians, two reptiles: Table 3) that potentially occur in Mexico City based on their distribution along the border with Morelos and the State of Mexico. We used distributional records in Vertnet.org and the Sistema Nacional de Información sobre Biodiversidad (SNIB-CONABIO) for the two neighboring states and Mexico City to generate this list. As more herpetological work is done near the borders with the neighboring states, we believe that these “likely to occur” species will be recorded for Mexico City.

### General distribution

Fifteen of the 18 species of amphibians found in Mexico City are endemic to Mexico. Two species are endemic to Mexico City (*Eleutherodactylus grandis* and *Ambystoma mexicanum*), one species has a spotty distribution in Mexico City and the State of Mexico, seven are distributed in central Mexico (*Dryophytes plicatus* [Brocchi, 1877], *Ambystoma altamirani* Dugès, 1895, *Aquiloeurycea cephalica* [Cope, 1865], *Chiropterotriton orculus* [Cope, 1865], *Pseudoeurycea altamontana* [Taylor, 1939], *P. leprosa* [Cope, 1869], and *P. tlilicxitl* Lara-Góngora, 2003), two are distributed in central Mexico and the Mexican Altiplano (*Rana montezumae* Baird, 1854, and *Anaxyrus compactilis* [Wiegmann, 1833]), one occurs in central Mexico, the Mexican Altiplano, the Sierra Madre Occidental, and the Sierra Madre Oriental (*Dryophytes eximius* [Baird, 1854]), and one occurs along the Neovolcanic Axis, the Sierra Madre Occidental, the Sierra Madre Oriental, and the Sierra Madre del Sur of Guerrero (*Isthmura belli* [Gray, 1850]). The three amphibian species not endemic to Mexico that inhabit Mexico City are species found in the United States and Mexico (Table 1). Twelve of the 14 species of lizards that occur in Mexico City are endemic to Mexico, six of the endemic species are restricted to a small area in the State of Mexico and Morelos and Mexico City, and in some cases the states of Puebla or Oaxaca (*Sceloporus aeneus* Wiegmann, 1828, *S. anahuacus* Lara-Góngora, 1983, *S. mucronatus* Cope, 1885, *S. palaciosi* Lara-Góngora, 1983, *S. sugillatus* Smith, 1942, and *Plestiodon copei* [Taylor, 1933]), and the other six endemics are widely distributed in the Neovolcanic Axis, including parts of the Sierra Madre Occidental, the Sierra Madre Oriental, and in some cases the Mexican Altiplano (*Barisia imbricata* [Wiegmann, 1828], *Phrynosoma orbiculare* [Linnaeus, 1758], *Sceloporus scalaris* Wiegmann, 1828, *S. spinosus* Wiegmann, 1828, *S. torquatus* Wiegmann, 1828, and *Plestiodon brevirostris* [Günther, 1860]). One of the non-endemic species ranges widely from southeastern United States, south through most of Mexico to southern Oaxaca (*Sceloporus grammicus* Wiegmann, 1828), and the other species ranges from central United States to Central America (*Aspidoscelis gularis* [Baird & Girard, 1852]) (Table 1). Twenty-one of the 29 species of snakes that inhabit Mexico City are endemic to Mexico. Of the eight snake species not endemic to Mexico that inhabit Mexico City, five are found in Canada and/or the United States and Mexico (*Diadophis punctatus* [Linnaeus, 1766], *Micrurus tener* Baird & Girard, 1853, *Rena dulcis* Baird & Girard, 1853, *Thamnophis eques* (Reuss, 1834), and *Crotalus molossus* Baird & Girard, 1853), one is found from Mexico to Central America (*Pituophis lineaticollis* [Cope, 1861]), and two range from the southern United States to Central America (*Senticolis triaspis* [Cope, 1866] and *Thamnophis cyrtopsis* [Kennicott, 1860]).

### Habitat types

The most diverse region of the city is the Forests and Ravines region, which is home to 43 species (13 amphibians, 30 reptiles), which represents 70.5% of the species pool for the city. This region occupies the second place in the territorial area of the city with 534.4 km^2^ (36.0% of the surface of Mexico City). This region is in the best conservation state and is where there is the possibility of rediscovering species known only from historical records such as *Geophis bicolor* Günther, 1868, *Geophis petersii* Boulenger, 1894, and *Rhadinaea taeniata* (Peters, 1863), or finding new species records such as for *Eleutherodactylus nitidus* (Peters, 1870), *Exerodonta smaragdina* (Taylor, 1940), and *Tantilla bocourti* (Günther, 1895). This region also has a high number of species listed in an IUCN protected category (7 amphibians, 1 reptile), listed in a SEMARNAT protected category (6 amphibians, 7 reptiles), and categorized as high risk by the EVS (7 amphibians, 15 reptiles). The second most diverse region of Mexico City is the Urban Parks and Gardens that hosts 34 species (13 amphibians, 21 reptiles). This region occupies the largest area of the city with 607.3 km^2^ (40.9% of the surface of Mexico City). It is also the most populated region and is dominated by urban habitats, which are generally not suitable for most amphibians and reptiles. However, it has two important urban parks, the Pedregal de San Angel Ecological Reserve (REPSA) and the Chapultepec Forest, where the largest number of species has been recorded for this region. The number of species in this region listed in an IUCN protected category (6 amphibians, 2 reptiles), listed as protected by SEMARNAT (6 amphibians, 7 reptiles), and categorized as high risk by the EVS (6 amphibians, 8 reptiles) is also high. The Wetlands of Xochimilco and Tláhuac and the Mountains of Xochimilco and Milpa Alta, and the Sierra de Guadalupe host similar numbers of amphibian and reptile species and also have similar numbers of species included in the IUCN and SEMARNAT lists or categorized as high risk by the EVS. In the Wetlands of Xochimilco and Tláhuac, 20 species (6 amphibians, 14 reptiles) are present, of which four are listed by the IUCN, five by SEMARNAT, and 10 are categorized as high risk by the EVS. In the Mountains of Xochimilco and Milpa Alta, 19 species (6 amphibians, 13 reptiles) are found, of which two are listed by the IUCN, four by SEMARNAT, and nine are categorized as high risk by the EVS. In the Sierra de Guadalupe, 23 species (6 amphibians, 17 reptiles) occur, of which one is listed by the IUCN, five by SEMARNAT, and eight are categorized as high risk by the EVS. The Sierra de Santa Catarina region is like an island, a mountain with disturbed vegetation surrounded by urban habitats. It has an area of only 31.4 km^2^ (2.1% of the area of Mexico City; Fig. 7) and harbors 13 species of reptiles. However, the government of Mexico City mentioned the presence of two amphibians and 14 reptiles, with only eight reptile species included in the SEMARNAT list (Gobierno del Distrito Federal [= Mexico City] 2005). None of the reptile species in this region are included in the IUCN list, three are in the SEMARNAT list, and four are categorized as high risk by the EVS. Our observations of the distribution of amphibians and reptiles in Mexico City are broadly consistent with the findings of García Vázquez and Méndez de la Cruz (2016) and García Vázquez et al. (2016).

### Conservation status

**Figure 8.**
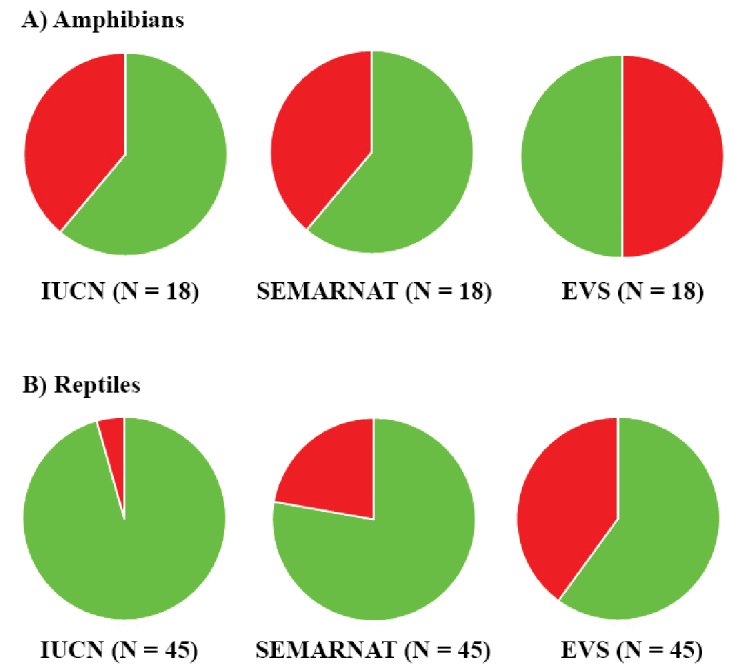
Percentage of species included in the IUCN Red List or listed by SEMARNAT, and the Environmental Vulnerability Score (EVS). **A** Amphibians **B** reptiles. Green is percentage in Data Deficient and Least Concern (IUCN); Not Listed and Subject to Special Protection (we regarded the category of Subject to Special Protection in SEMARNAT equivalent to IUCN’s Least Concern) (SEMARNAT); low and medium EVS. Red is percentage in protected categories or high EVS. *N* is the number of species assessed by each agency.

Eleven of the 63 species (17.5%) of amphibians and reptiles in Mexico City are included in the IUCN Red List (i.e., Vulnerable, Near Threatened, or Endangered), 17 (27.0%) are placed in a protected category (excluding NL and Pr, this last category is equivalent to the LC category of IUCN) by SEMARNAT, and 27 species (42.9%) are categorized as high risk by the EVS (Fig. 8; Table 2). For amphibians, 38.9% (7 of 18 species) are included in the IUCN Red List and are protected by SEMARNAT, and 50.0% (9 species) are at high risk according to the EVS (Fig. 8; Table 2). For reptiles, 4.4% (2 of 45 species) are listed by the IUCN, 22.2% (10 species) are protected by SEMARNAT, and 40.0% (18 species) are at high risk according to the EVS (Fig. 8; Table 2). These results suggest that the amphibians of Mexico City are considered to be of relatively high conservation concern at a global and national scale (IUCN and SEMARNAT lists), but there are even greater conservation concerns based on the EVS. The limited distribution of half of the amphibian species that inhabit Mexico City, coupled with the loss of available habitat, places them in a delicate conservation status. For example, the Axolotl, *Ambystoma mexicanum*, which is listed as Critically Endangered by the IUCN and In Danger of Extinction by SEMARNAT, currently appears to be limited to Lake Xochimilco and faces threats such as introduced predators, illegal collection, and pollution (Griffiths et al. 2004; Contreras et al. 2009; Recuero et al. 2010). On the other hand, reptiles are of lesser conservation concern than amphibians according to the IUCN and SEMARNAT lists, but not the EVS list (Fig. 8), which is a reflections that EVS uses more variables in determining country-level conservation status than either the IUCN or SEMARNAT (see Wilson et al. 2013a, 2013b).

**Table 2.** Summary of native species present in Mexico City by class, family, order, and suborder. Status summary indicates the number of species found in each IUCN conservation status in the order DD, LC, VU, NT, EN, CR (see Table 1 for abbreviations). For mean EVS (Environmental Vulnerability Score), scores ≥14 are considered to have high vulnerability (Wilson et al. 2013a, b). Conservation status in Mexico are number of species in each category (NL, Pr, A, P; see Table 1 for abbreviations) according to SEMARNAT (2019).

Scientific name	genera	species	IUCN	x̄ EVS	SEMARNAT
**Class Amphibia**			**DD, LC, VU, NT, EN, CR**		**NL, Pr, A, P**
**Order Anura**	**6**	**9**	**0, 7, 0, 0, 0, 2**	**11**	**5, 2, 1, 1**
Bufonidae	1	1	0, 1, 0, 0, 0, 0	14	1, 0, 0, 0
Craugastoridae	1	1	0, 1, 0, 0, 0, 0	8	1, 0, 0, 0
Eleutherodactylidae	1	1	0, 0, 0, 0, 0, 1	18	0, 1, 0, 0
Hylidae	1	3	0, 3, 0, 0, 0, 0	9.3	2, 0, 1, 0
Ranidae	1	2	0, 1, 0, 0, 0, 1	14	0, 1, 0, 1
Scaphiopodidae	1	1	0, 1, 0, 0, 0, 0	3	1, 0, 0, 0
**Order Caudata**	**5**	**9**	**0, 2, 2, 1, 3, 1**	**14.7**	**2, 2, 4, 1**
Ambystomatidae	1	3	0, 1, 0, 0, 1, 1	12.7	0, 1, 1, 1
Plethodontidae	4	6	0, 1, 2, 1, 2, 0	15.7	2, 1, 3, 0
**Subtotal**	**11**	**18**	**0, 9, 2, 1, 3, 3**	**12.8**	**7, 4, 5, 2**
**Class Reptilia**					
**Order Squamata**	**19**	**43**	**2, 39, 1, 0, 1, 0**	**12.3**	**23, 10, 9, 1**
**Suborder Lacertilia**	**5**	**14**	**0, 14, 0, 0, 0, 0**	**12.6**	**10, 3, 1, 0**
Anguidae	1	1	0, 1, 0, 0, 0, 0	14	0, 1, 0, 0
Phrynosomatidae	2	10	0, 10, 0, 0, 0, 0	12.8	8, 1, 1, 0
Scincidae	1	2	0, 2, 0, 0, 0, 0	12.5	1, 1, 0, 0
Teiidae	1	1	0, 1, 0, 0, 0, 0	9	1, 0, 0, 0
**Suborder Serpentes**	**14**	**29**	**2, 25, 1, 0, 1, 0**	**12.2**	**13, 7, 8, 1**
Colubridae	6	9	0, 9, 0, 0, 0, 0	11	5, 2, 2, 0
Dipsadidae	3	5	2, 3, 0, 0, 0, 0	11.8	3, 2, 0, 0
Elapidae	1	1	0, 1, 0, 0, 0, 0	11	1, 0, 0, 0
Leptotyphlopidae	1	1	0, 1, 0, 0, 0, 0	13	1, 0, 0, 0
Natricidae	2	7	0, 5, 1, 0, 1, 0	12.1	2, 0, 5, 0
Viperidae	1	6	0, 6, 0, 0, 0, 0	14.5	1, 3, 1, 1
**Order Testudines**	**1**	**2**	**0, 2, 0, 0, 0, 0**	**10.5**	**0, 2, 0, 0**
Kinosternidae	1	2	0, 2, 0, 0, 0, 0	10.5	0, 2, 0, 0
**Subtotal**	**20**	**45**	**2, 41, 1, 0, 1, 0**	**12.2**	**23, 12, 9, 1**
**Total**	**31**	**63**	**2, 50, 3, 1, 4, 3**	**12.4**	**30, 16, 14, 3**

**Table 3.** List of amphibian and reptile species that potentially occur in Mexico City.

Taxon	Likely to occur in:
**Class Amphibia**	
**Order Anura**	
**Family Bufonidae**	
*Incilius marmoreus* (Wiegmann, 1833)	southern Mexico City
*Incilius occidentalis* (Camerano, 1879)	southern Mexico City
**Family Eleutherodactylidae**	
*Eleutherodactylus nitidus* (Peters, 1870)	southern Mexico City
**Family Hylidae**	
*Exerodonta smaragdina* (Taylor, 1940)	southern Mexico City
*Tlalocohyla smithii* (Boulenger, 1902)	southern Mexico City
**Family Ranidae**	
*Rana spectabilis* Hillis & Frost, 1985	southern Mexico City
**Class Reptilia**	
**Order Squamata**	
**Suborder Lacertilia**	
**Family Scincidae**	
*Plestiodon indubitus* (Taylor, 1933)	southern Mexico City
**Suborder Serpentes**	
**Family Colubridae**	
*Tantilla bocourti* (Günther, 1895)	southern Mexico City

### Comparison with neighboring states

Mexico City shares more than 90% of its species (59 of 63 species = 93.7%, Table 4), with the State of Mexico, such that the herpetofauna of Mexico City is practically contained in that of the State of Mexico. Both the city and state are in the Basin of Mexico, which is included in the physiographic province of the Neovolcanic Axis, and all of Mexico City belongs to the sub-province of Lagos y Volcanes de Anáhuac, which also forms a part of the State of Mexico. Thus, the topographic and physiographic characteristics shared by these two entities result in a great similarity of their biological diversity, including their herpetofaunas. Additionally, the State of Mexico surrounds Mexico City on three sides (west, north, and east), and this, coupled with the small territorial area of Mexico City, results in a nesting of the species richness of Mexico City in the species richness of the State of Mexico. There are four species that Mexico City does not share with the State of Mexico. These include *Eleutherodactylus grandis*, which is limited to the Pedregal de San Angel in the Urban Parks and Gardens region; *Ambystoma mexicanum*, currently limited to two wild populations, one in the channels of Lake Xochimilco and a second in the remnants of Lake Chalco, both in southern Mexico City, where it faces serious conservation problems (Zambrano-González et al. 2003; Recuero et al. 2010); *Geophis petersi*, a snake with secretive habits, which is difficult to find and known only from the type series; and *Rena dulcis*, reported by Méndez de la Cruz et al. (2007, 2009) in the Pedregal de San Angel.

*Geophis petersi* was collected by H. Doorman in 1868 and its type locality at Mexico City was questioned and restricted to Pátzcuaro, Michoacán by Smith and Taylor (1950); however, Downs (1967) pointed out that this restriction was unjustified and that the presence of *G. petersi* at the southern tip of the Mexican Altiplano seems reasonable. Due to the secretive habits and presence of similar habitats in the State of Mexico, it is possible that *G. petersi* also inhabits the State of Mexico but not yet recorded from there. A similar situation occurs for *R. dulcis*, the other species not yet recorded in the State of Mexico, but due to its secretive habits and tiny size, this snake might also likely occur in the State of Mexico.

Mexico City shares 49 of its amphibians and reptiles with Morelos (77.8%; Table 4). The lower percentage of shared species compared to the State of Mexico is partly due to the fact that Morelos is not within the Basin of Mexico; however, part of it is included in the physiographic province of the Neovolcanic Axis, subprovince of Lagos y Volcanes de Anáhuac. In addition, less temperate forest is present in Morelos, and Morelos is smaller compared to the State of Mexico (Lemos-Espinal and Smith 2020). These characteristics result in fewer species shared with Morelos than with the State of Mexico. Seven of the 14 species that Mexico City does not share with Morelos probably inhabit this latter state, but they have not been recorded yet (*Ambystoma velasci*, *Sceloporus anahuacus*, *Diadophis punctatus*, *Geophis bicolor*, *G. petersi*, *Thamnophis melanogaster*, and *T. pulchrilatus*) (Lemos-Espinal and Smith 2020).

**Table 4.** Summary of the numbers of species shared between Mexico City and neighboring Mexican states (not including introduced species). The percent of Mexico City species shared by a neighboring state are given in parentheses. Total refers to the total number of species found in Mexico City and two neighboring states (i.e., regional species pool) and the number in parentheses in this column is the percent of the regional species pool found in Mexico City. – indicates either Mexico City or the neighboring state has no species in the taxonomic group, or none of that specific taxon is shared between the states, thus no value for shared species is provided.

Taxon	Mexico City	State of Mexico	Morelos	Total
**Class Amphibia**	**18**	**16 (88.9)**	**14 (77.8)**	**55 (32.7)**
**Order Anura**	**9**	**8 (88.9)**	**7 (77.8)**	**39 (23.1)**
Bufonidae	1	1 (100)	1 (100)	5 (20.0)
Centrolenidae	–	–	–	1 (0)
Craugastoridae	1	1 (100)	1 (100)	5 (20.0)
Eleutherodactylidae	1	–	–	5 (20.0)
Hylidae	3	3 (100)	3 (100)	10 (30.0)
Leptodactylidae	–	–	–	1 (0)
Microhylidae	–	–	–	3 (0)
Phyllomedusidae	–	–	–	1 (0)
Ranidae	2	2 (100)	1 (50)	7 (28.6)
Scaphiopodidae	1	1 (100)	1 (100)	1 (100)
**Order Caudata**	**9**	**8 (88.9)**	**7 (77.8)**	**16 (56.3)**
Ambystomatidae	3	2 (66.7)	1 (33.3)	8 (37.5)
Plethodontidae	6	6 (100)	6 (100)	8 (75.0)
**Class Reptilia**	**45**	**43 (95.6)**	**35 (77.8)**	**125 (36.0)**
**Order Squamata**	**43**	**41 (95.3)**	**33 (76.7)**	**120 (35.8)**
**Suborder Lacertilia**	**14**	**14 (100)**	**12 (85.7)**	**49 (28.6)**
Anguidae	1	1 (100)	1 (100)	5 (20.0)
Dactyloidae	–	–	–	1 (0)
Eublepharidae	–	–	–	1 (0)
Helodermatidae	–	–	–	1 (0)
Iguanidae	–	–	–	1 (0)
Phrynosomatidae	10	10 (100)	9 (90.0)	23 (43.5)
Phyllodactylidae	–	–	–	3 (0)
Scincidae	2	2 (100)	2 (100)	6 (33.3)
Teiidae	1	1 (100)	–	8 (12.5)
**Suborder Serpentes**	**29**	**27 (93.1)**	**21 (72.4)**	**71 (40.8)**
Boidae	–	–	–	1 (0)
Colubridae	9	9 (100)	9 (100)	24 (37.5)
Dipsadidae	5	4 (80.0)	2 (40.0)	18 (27.8)
Elapidae	1	1 (100)	1 (100)	3 (33.3)
Leptotyphlopidae	1	–	–	3 (33.3)
Loxocemidae	–	–	–	1 (0)
Natricidae	7	7 (100)	4 (57.1)	10 (70.0)
Viperidae	6	6 (100)	5 (83.3)	11 (54.5)
**Order Testudines**	**2**	**2 (100)**	**2 (100)**	**5 (40.0)**
Emydidae	–	–	–	1 (0)
Geoemydidae	–	–	–	1 (0)
Kinosternidae	2	2 (100)	2 (100)	3 (66.7)
**Total**	**63**	**59 (93.7)**	**49 (77.8)**	**180 (35.0)**

## Appendix 1

Museum collections included in the VertNet.org database records of Mexico City amphibians and reptiles that house specimens of the first record of a species in Mexico City.

**AMNH** Collection of Herpetology, Herpetology Department, American Museum of Natural History

**BMNH** Zoological Collection, Natural History Museum (London)

**CNAR** Colección Nacional de Anfibios y Reptiles, Instituto de Biología, Universidad Nacional Autónoma de México

**ENCB** Escuela Nacional de Ciencias Biológicas, Instituto Politécnico Nacional

**FMNH** Division of Amphibians and Reptiles, Field Museum of Natural History

**MCZ** Collection of Herpetology, Museum of Comparative Zoology, Harvard University Cambridge

**MVZ** Museum of Vertebrate Zoology at Berkeley, Herpetological Collection

**MZFC** Colección Herpetológica, Museo de Zoología Alfonso L. Herrera, Facultad de Ciencias, UNAM

**ROM** Herpetology Collection, Royal Ontario Museum

**UMMZ** Collection of Herpetology, Museum of Zoology, University of Michigan Ann Arbor

**USNM** Collection of Herpetology, Department of Vertebrate Zoology, National Museum of Natural History, Smithsonian Institution
